# Improved production of fatty alcohols in cyanobacteria by metabolic engineering

**DOI:** 10.1186/1754-6834-7-94

**Published:** 2014-06-18

**Authors:** Lun Yao, Fengxia Qi, Xiaoming Tan, Xuefeng Lu

**Affiliations:** 1 Key Laboratory of Biofuels, Shandong Provincial Key Laboratory of Energy Genetics, Qingdao Institute of Bioenergy and Bioprocess Technology, Chinese Academy of Sciences, No. 189 Songling Road, Qingdao 266101, China; 2 University of Chinese Academy of Sciences, No. 19 jia, Yuquan Street, Shijingshan District, Beijing 100049, China

**Keywords:** Fatty alcohol, Cyanobacteria, Fatty acyl-CoA reductase, *Marinobacter aquaeolei* VT8

## Abstract

**Background:**

Fatty alcohols are widely used in industrial chemicals. The biosynthetic pathways for fatty alcohols are diverse and widely existing in nature. They display a high capacity to produce fatty alcohols by the metabolic engineering of different microbe hosts. Direct recycling of carbon dioxide to fatty alcohols can be achieved by introducing a fatty alcohol-producing pathway into photosynthetic cyanobacteria. According to our precious reports, a relatively low yield of fatty alcohols was obtained in the genetically engineered cyanobacterium *Synechocystis* sp. PCC 6803.

**Results:**

The photosynthetic production of fatty alcohols in *Synechocystis* sp. PCC 6803 was improved through heterologously expressing fatty acyl-Coenzyme A (acyl-CoA) reductase gene *maqu_2220* from the marine bacterium *Marinobacter aquaeolei* VT8. Maqu_2220 has been proved to catalyze both the four-electron reduction of fatty acyl-CoA or acyl-Acyl Carrier Protein (acyl-ACP) and the two-electron reduction of fatty aldehyde to fatty alcohol. The knockout of the aldehyde-deformylating oxygenase gene (*sll0208*) efficiently blocked the hydrocarbon accumulation and redirected the carbon flux into the fatty alcohol-producing pathway. By knocking-out both *sll0208* and *sll0209* (encoding an acyl-ACP reductase), the productivity of fatty alcohols was further increased to 2.87 mg/g dry weight.

**Conclusions:**

The highest yield of fatty alcohols was achieved in cyanobacteria by expressing the prokaryotic fatty acyl-CoA reductase Maqu_2220 and knocking-out the two key genes (*sll0208* and *sll0209*) that are involved in the alka(e)ne biosynthesis pathway. Maqu_2220 was demonstrated as a robust enzyme for producing fatty alcohols in cyanobacteria. The production of fatty alcohols could be significantly increased by blocking the hydrocarbon biosynthesis pathway.

## Background

As concerns over the fuel shortage and environmental problems caused by the use of petroleum-based fuels, the development of biofuels has been considered as a promising choice to replace or supplement traditional fossil fuels. Numerous reviews have been published focusing on this area and various kinds of biofuels, including fatty acid-based biofuels, have been explored [1-6].

Cyanobacteria are non-food-based feedstock resources that can use non-productive land and water sources. They are able to convert solar energy and CO_2_ for the synthesis of co-products and do not lead to loss of ecosystems [7]. The production of biofuels and biochemicals in cyanobacteria are receiving increasing attention because of their photosynthetic capability and genetic engineering capacity. The genetic manipulation platform for cyanobacteria is well established and over 126 genomes of cyanobacterial strains have already been sequenced [8]. Several reviews have been published to illustrate the advantages and challenges in the engineering of cyanobacteria to produce valuable products [9,10]. Up to now, different kinds of molecules such as hydrogen, ethanol, isobutyraldehyde, isoprene, β-caryophyllene, sucrose, butanol, fatty alcohols, and fatty acids have been produced in cyanobacteria [11-21]. The activation and recycling of fatty acids in cyanobacteria has also been investigated [22,23]. In 2010, an alkane biosynthesis pathway that consists of an acyl-Acyl carrier protein reductase (AAR) and an aldehyde-deformylating oxygenase (ADO) has been identified in cyanobacteria [24]. A side pathway responsible for a one-step conversion of fatty aldehyde to free fatty acid by an aldehyde dehydrogenase (Orf0489) was also reported in the cyanobacterium *Synechococcus elongatus* PCC 7942 [25]. Its ortholog Slr0091 was also identified in the cyanobacterium strain *Synechocystis* sp. PCC 6803 (hereafter *Synechocystis*) [26].

**Figure 1:**
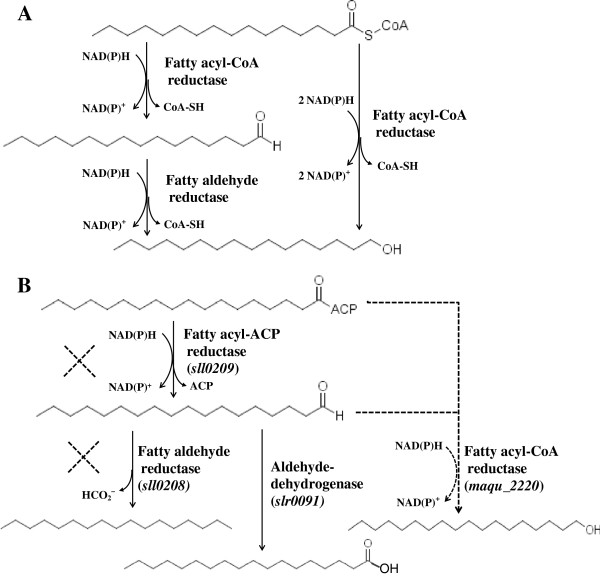
**Fatty alcohol biosynthesis pathways. (A)** Two different reaction schemes of fatty alcohol biosynthesis. Fatty alcohol could be synthesized via consecutive reduction reactions of a fatty acyl-CoA reductase and a fatty aldehyde reductase or via a single-step reduction of fatty acyl-CoA reductase. **(B)** Schematic view of fatty alcohol biosynthesis pathways in *Synechocystis* sp. PCC 6803 (*Synechocystis)*. Solid lines show the native alka(e)ne and free fatty acid biosynthesis pathways in *Synechocystis*; dashed lines show the constructed fatty alcohol biosynthesis pathways in *Synechocystis* by knocking out the endogenous *sll0208*/*sll0209* gene and heterologously expressing the fatty acyl-CoA reductase (*maqu_2220*) from *Marinobacter aquaeolei* VT8.

Fatty alcohols are important industrial chemicals. They can also be good biofuel additives because of their perfect fuel properties. In nature, fatty alcohols exist as free fatty alcohols or waxes (oxygen esters of primary fatty alcohols and fatty acids) in a wide range of organisms including bacteria, plants, and mammals [27-33]. Generally, two different reaction schemes can generate primary fatty alcohols in biological systems (Figure 1A). One scheme is the four-electron reduction of fatty acyl-CoA (or acyl-ACP) to the corresponding fatty alcohol [27]. The other scheme involves two distinct enzymes: a fatty aldehyde forming acyl-CoA reductase which catalyses a two-electron reduction of fatty acyl-CoA to a fatty aldehyde intermediate, and a fatty aldehyde reductase which catalyses the reduction of fatty aldehyde to fatty alcohol [27,34]. Thus, the fatty acyl-CoA (or acyl-ACP) reductases (FARs) can be divided into two general types in terms of their reaction schemes: the four-electron and two-electron reduction FARs. Additional file 1: Table S1 lists the reported FARs from different organisms as well as their substrate specificities, and types. The phylogenetic relationships of these enzymes were displayed as an unrooted tree generated by the neighbor-joining method (Additional file 2: Figure S1).

Wahlen *et al*. [35] characterized an enzyme (Maqu_2220) performing reduction of fatty aldehydes to fatty alcohols from *Marinobacter aquaeolei* VT8 (hereafter *M. aquaeolei* VT8). They designated Maqu_2220 a fatty aldehyde reductase (FALDR) since it could convert fatty aldehydes to fatty alcohols through an *in vitro* experiment [35]. Later on, Hofvander *et al*. performed another *in vitro* assay and contradictorily reported that Maqu_2220 could not only catalyze the conversion of fatty aldehydes to fatty alcohols, but also catalyze the direct reduction of fatty acyl-CoAs (or acyl-ACPs) to fatty alcohols [36]. *In vivo*, however, it is still not clear which scheme is the major contributor for fatty alcohol synthesis by Maqu_2220. The gene *maqu_2220* was expressed in *Escherichia coli (E. coli)* and a remarkable level of fatty alcohols (1.725 g/L) were produced in an optimized mutant strain of *E. coli* under fermentation conditions [37]. In a recent paper, *Saccharomyces cerevisiae* was also engineered to produce fatty alcohols (almost 100 mg/L in 168 hours) by overexpressing the mouse fatty acyl-CoA reductase (mFAR1) [38].

Cyanobacteria do not have native fatty alcohol synthesis pathways. The production of fatty alcohols in cyanobacteria was investigated in our previous study by heterologously expressing the fatty acyl-CoA reductase (FAR) from mouse, *jojoba* (*Simmondsia chinensis*), and *Arabidopsis* (*Arabidopsis thaliana*). Up to 9.73 ± 2.73 μg OD_730_^−1^ L^−1^ of fatty alcohol was obtained in the *Synechocystis* strain expressing the *jojoba* FAR gene [15]. The yield of fatty alcohol in cyanobacteria was then increased to approximately 761 ug/g dry mass by blocking the glycogen synthesis and poly-3-hydroxybutyrate (PHB) synthesis as well as by over-expressing the FAR genes [39]. However, compared with *E. coli* and yeast, the construction of fatty alcohol biosynthetic pathway in cyanobacteria is still in its infancy. Thus, more genetic engineering work is needed to improve the cyanobacterial fatty alcohol production, such as the introduction of the novel FAR genes into cyanobacterial hosts or the disruption of the competitive pathway.

In this study, the *maqu_2220* gene was heterologously expressed in *Synechocystis* for the production of fatty alcohols. In order to improve the fatty alcohol synthesis, the acyl-ACP reductase (encoded by *sll0209*) and aldehyde-deformylating oxygenase (encoded by *sll0208*) in the fatty alkane biosynthesis pathway were deleted in the *maqu_2220-*expressing *Synechocystis* strain (Figure 1B). These approaches resulted in a significant increase of cyanobacterial fatty alcohol production, which reached up to 2.87 mg/g dry weight.

## Results and discussion

### Production of fatty alcohols by heterologously expressing the *maqu_2220* gene in *Synechocystis*

**Figure 2:**
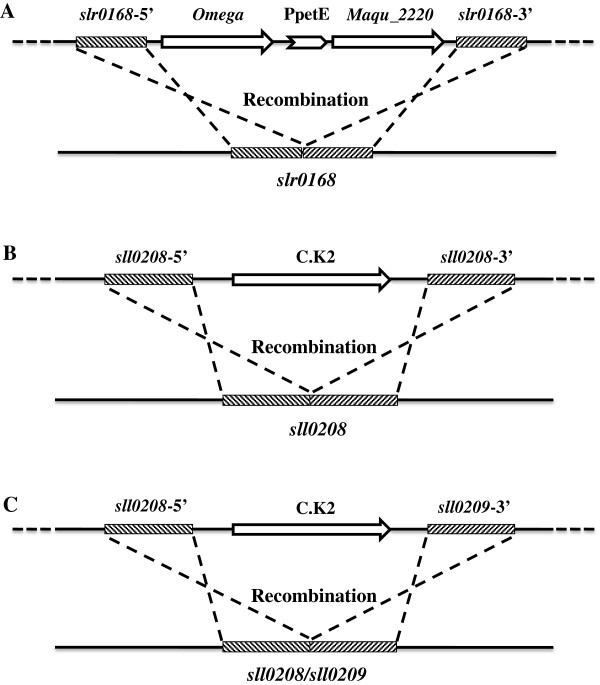
**Expression of the *maqu_2220 *gene and disruption of the *sll0208*/*sll0209 *gene in *Synechocystis. *(A)** Schematic representation of recombination to integrate the *maqu_2220* gene expression cassette into *slr0208* locus. *slr0168*-5' (or *slr0168*-3'), the 5' (or 3') end of *slr0168* that was used for integrating the expressing cassette into the *slr0168* locus by double homologous recombination. *Omega*, spectinomycin resistance gene. **(B)** Disruption of *sll0208* in *Synechocystis. sll0208*-5' (or *sll0208*-3'), the 5' (or 3') end of *sll0208* gene used for integrating the kanamycin resistance gene into the *sll0208* locus by double homologous recombination. C.K2, the gene cassette containing a kanamycin resistance gene. **(C)** Disruption of both *sll0208* and *sll0209* in *Synechocystis.* The 5' end of *sll0208* and the 3' end of *sll0209* (*sll0209*-3') were used for integrating the kanamycin resistance gene into the *sll0208* and the *sll0209* locus by double homologous recombination. C.K2, the gene cassette containing a kanamycin resistance gene. P_petE_, the promoter of the *petE* gene.

**Figure 3:**
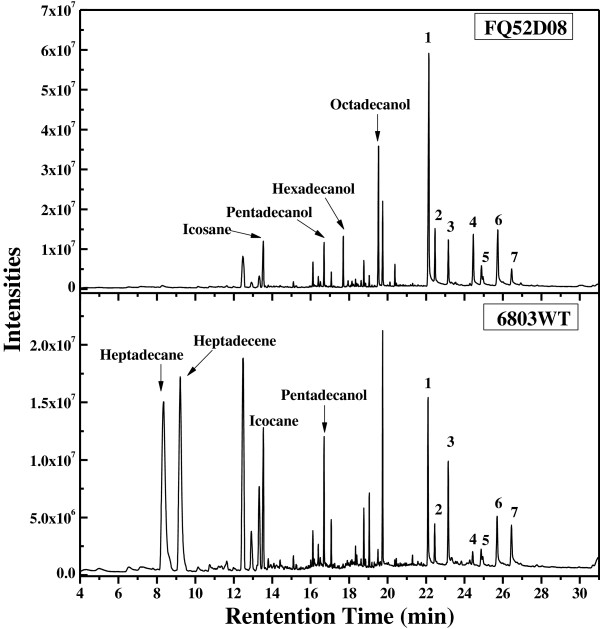
**GC-MS analysis of alkanes, fatty alcohols and free fatty acids in wild-type *Synechocystis *and Syn-FQ52D08.** Peaks: (1) palmitic acid; (2) palmitoleic acid; (3) heptadecanoic acid; (4) stearic acid; (5) oleic acid; (6) linoleic acid; (7) linolenic acid. Eicosane, pentadecanol, and heptadecanoic acid were used as internal standards for the analysis of alkanes, fatty alcohols, and free fatty acids respectively. 6803WT: the wild-type strain of *Synechocystis*; Syn-FQ52D08: the strain with deletion of *sll0208* and expression of *maqu_2220*.

**Figure 4:**
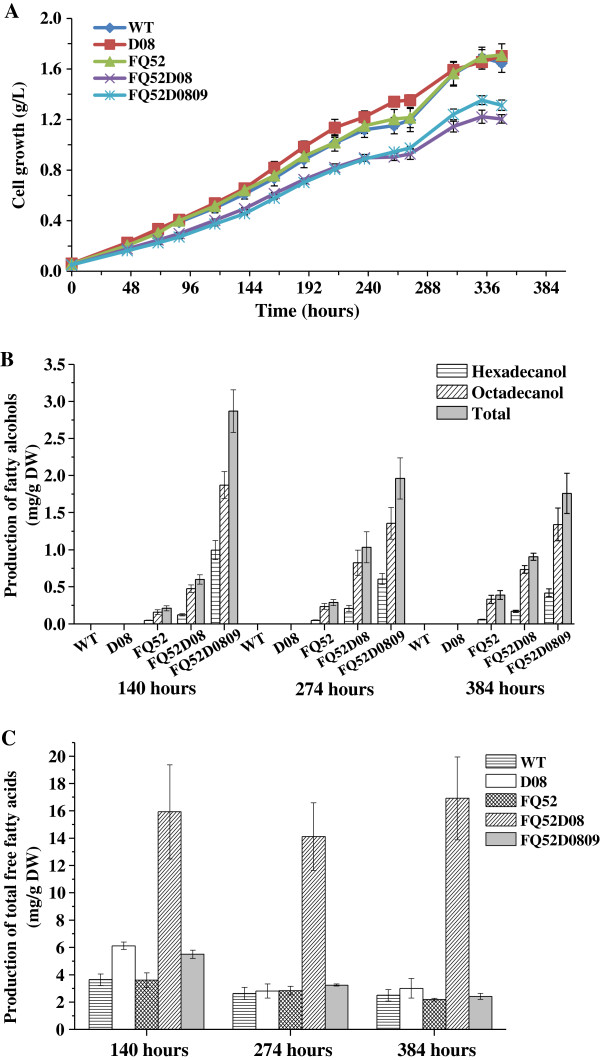
**Contents and composition of fatty acid derivatives in the mutant and the wild-type *Synechocystis. *(A)** Growth curves of WT, Syn-D08, Syn-FQ52, Syn-FQ52D08, and Syn-FQ52D0809; **(B)** Fatty alcohol production in the *Synechocystis* mutant strains; **(C)** Total free fatty acid production in *Synechocystis* WT and mutant strains. Error bars indicate standard deviation (n = 3). WT: the wild-type strain of *Synechocystis*; Syn-D08: the strain with deletion of *sll0208*; Syn-FQ52: the strain with expression of *maqu_2220*; Syn-FQ52D08: the strain with deletion of *sll0208* and expression of *maqu_2220*; Syn-FQ52D0809: the strain with expression of *maqu_2220* and double knockout of *sll0208* and *sll0209*.

The *maqu_2220* gene from *M. aquaeolei* VT8 was expressed under the control of the promoter P_petE_ (Nucleotide sequences listed in the Additional file 3); the Omega cassette [40] which harbors a spectinomycin resistance gene was used as a selection marker (Figure 2A). The mutant strain Syn-FQ52 was obtained by transformation of *Synechocystis* with the plasmid pFQ52. The expressing cassette was integrated into the *slr0168* locus of the genome by double homologous recombination. The integration of the expressing cassette and the complete segregation of the mutant strain were confirmed by PCR analysis (see Additional file 4: Figure S2). The most abundant fatty alcohols in Syn-FQ52 are hexadecanol and octadecanol, which are the same as those in the strain expressing the *jojoba* FAR [15]. Syn-FQ52 was able to produce fatty alcohols with a yield of 0.21 ± 0.03 mg/g Dry Weight (DW), 0.29 ± 0.04 mg/g DW and 0.39 ± 0.06 mg/g DW in total after culturing for 140, 274, and 384 hours, respectively. No fatty alcohol was detected in the wild-type *Synechocystis* strain (Figures 3 and 4B).

The bacterial FAR Maqu_2220 was shown to have broad substrate activity towards fatty acyl-CoAs and fatty aldehydes with different carbon chain lengths and unsaturation [35,36]. However, no unsaturated fatty alcohols were detected in our *maqu_2220*-expressing strains of *Synechocystis*. It suggests that the profile of fatty alcohols not only depends on the substrate specificity of FARs but can also be strongly influenced by the host organism.

### Deletion of the aldehyde-deformylating oxygenase gene from the hydrocarbon biosynthesis pathway affected the alka(e)ne and fatty acids production

The ADO gene *sll0208* in both the wild-type *Synechocystis* and Syn-FQ52 was inactivated by inserting a kanamycin resistance gene in the middle of the *sll0208* gene (Figure 2B), generating Syn-D08 and Syn-FQ52D08, respectively. The gene replacement was confirmed by PCR assay. Only a single 3.3 kb-band was amplified from the genomic DNA of the *sll0208* mutant strain. The band size is not the same as that in the wild-type control (see Additional file 4: Figure S2). This indicates that the *sll0208* mutant was completely segregated and the *sll0208* gene was completely disrupted.

Syn-FQ52 showed similar fatty alka(e)ne and free fatty acid accumulations as the wild-type *Synechocystis* (Additional file 5: Figure S3 and Figure 4C). Fatty alka(e)nes took a large proportion of all the fatty acid derivatives in *Synechocystis* (Additional file 5: Figure S3C and D). Heptadecane and heptadecene were the most abundant hydrocarbons in the wild-type *Synechocystis*. By contrast, there were no hydrocarbons detected in Syn-D08 and Syn-FQ52D08, indicating that the alka(e)ne biosynthesis pathway was completely inactivated by the disruption of *sll0208*.

As the hydrocarbon synthesis was blocked, a large amount of fatty aldehydes was supposed to accumulate in the *sll0208* deletion strains. However, no fatty aldehyde was detected (Additional file 6: Figure S4) in Syn-D08 and Syn-FQ52D08 based on the extraction and Gas chromatography–mass spectrometry (GC-MS) method in this study. As controls, the pure fatty aldehydes proved to be quite stable under the same extraction and analysis procedures (Additional file 7: Figure S5).

One possibility is that the generated fatty aldehydes were oxidized by the newly discovered aldehyde dehydrogenase in cyanobacteria [26,41]. Kaiser *et al.* detected fatty aldehyde in *S. elongatus* PCC 7942 with over-expression of the fatty acyl-ACP reductase (encoded by *orf1594*) and deletion of the aldehyde dehydrogenase (encoded by *orf0489*) [41]. The ortholog of Orf0489 in *Synechocystis*, Slr0091, had also been proved to possess the activity of converting fatty aldehyde to fatty acids [26]. Thus, there is a chance that the fatty aldehydes accumulated in Syn-D08 and Syn-FQ52D08 were efficiently converted to fatty acids by the aldehyde dehydrogenase. In accordance with this, the total free fatty acids in Syn-D08 and Syn-FQ52D08 increased dramatically compared to WT strain (Figure 4C). Further, as an intermediate of the fatty acid pathway, fatty aldehydes are probably toxic for cyanobacteria, thus cyanobacteria may only possess a low level of intracellular fatty aldehyde pools.

### The enhanced production of fatty alcohols in *Synechocystis* by the deletion of the *sll0208* gene

By inactivation of the *sll0208* gene, no hydrocarbon was detected and the metabolic flux was redirected to the synthesis of fatty alcohols. Figure 4B shows the yield of fatty alcohol in the mutant and wild type strains of *Synechocystis* culturing for 140, 274 and 384 hours. The yield of fatty alcohols in Syn-FQ52D08 showed a significant increase (2.8, 3.6, and 2.3-fold of that in Syn-FQ52 at 140, 274, and 384 hours, respectively), reaching up to 0.6 ± 0.06, 1.03 ± 0.21, and 0.91 ± 0.05 mg/g DW (Figure 4B). The fatty alcohol productivity reported here had a significant improvement compared to that in Syn-XT14 (9.73 ± 2.73 μg OD_730_^−1^ L^−1^) as previously published by our lab [15].

The free fatty acids (FFA) content was also increased in Syn-FQ52D08 (Figure 4C), which was about 3.4 times, 4 times and 7 times higher compared to that of Syn-FQ52 respectively. In contrast, the FFA content in Syn-D08 was only slightly increased compared to that of the wild-type *Synechocystis* (Figure 4C). More obviously, the total fatty acyl chains (fatty alcohol, hydrocarbon, and free fatty acid) in Syn-FQ52D08 were much more than that in Syn-FQ52 and Syn-D08 (Additional file 5: Figure S3D). It suggests that more carbon flux could be driven into the fatty acid biosynthesis pathway by expressing the Maqu_2220 and disruption of the *sll0208* gene in Syn-FQ52D08.

### A further increase in fatty alcohol production by double knock-out of *sll0208* and *sll0209*

Even though Maqu_2220 from *M. aquaeolei* VT8 has been proved to catalyze both the reduction of fatty acyl-CoA (or acyl-ACP) to fatty alcohols and the reduction of fatty aldehydes to fatty alcohols [35,36], it was unclear which pathway (Figure 1B) contributes to the increase of fatty alcohols in the mutant strain Syn-FQ52D08. The mutant strain Syn-FQ52D0809 was thereby constructed by inactivation of both the *sll0208* and *sll0209* gene in Syn-FQ52 (Figure 2C).

Figure 4A compared the growth of the mutant and the wild type strains of *Synechocystis*. It can be found that the Syn-D08 and Syn-FQ52 showed a similar growth trend as the wild type strain, while the mutant Syn-FQ52D08 and Syn-FQ52D0809 exhibited the slightly lower growth rate. Figure 4B showed a further increase in fatty alcohol production by deletion of both the *sll0208* and the *sll020*9 gene. The fatty alcohol yield in Syn-FQ52D0809 was 2.87 ± 0.29, 1.96 ± 0.28, and 1.76 ± 0.27 mg/g DW after 140, 274 and 384 hours’ cultivation, respectively (5, 1.9, and 1.93-fold of the fatty alcohol yield in Syn-FQ52D08, respectively). Except for inactivation of the fatty alka(e)ne synthesis, the deletion of *sll0209* would block the fatty aldehydes synthesis as well as its downstream fatty acids synthesis that is catalyzed by the fatty aldehyde dehydrogenase. The enhanced production of fatty alcohols could be attributed to the increase of the fatty acyl-CoA (or acyl-ACP) pool in *Synechocystis* by the inactivation of *sll0208* and *sll0209*.

Syn-FQ52, Syn-FQ52D08, and Syn-FQ52D0809 showed different trends of the fatty alcohol productivity over the three time points. The yield of fatty alcohols of Syn-FQ52 showed a continuous increase during the 16 days of culturing, while the fatty alcohol productivity in Syn-FQ52D0809 decreased as the cell growth increased. Syn-FQ52D08 showed a different trend in fatty alcohol productivity. It was 0.6 ± 0.06 mg/g DW at 140 hours and subsequently reached a maximum at 274 hours (1.03 ± 0.21 mg/g DW), followed by a slight decrease at 384 hours (0.91 ± 0.05 mg/g DW). This indicates that fatty alcohol synthesis is related with the genotype of the strains and their growth phases. Furthermore, we also found that the newly transformed strains provided better fatty alcohol production. These results indicate that unknown mechanisms may exist for the regulation of the heterologous fatty alcohol synthesis in cyanobacteria.

The proportion of fatty acids, fatty alkane, and fatty alcohols in all strains were compared in Additional file 5: Figure S3. In strain Syn-FQ52D0809, the fatty alcohols comprised approximately 40% of the fatty acyl chains (Additional file 5: Figure S3C). In another aspect, however, it was found that the deletion of *sll0208* can dramatically raise the percentage of fatty acids. For instance, Syn-D08 mainly produced approximately 99% fatty acids, while Syn-FQ52D08 produced approximately 95% fatty acids and 5% fatty alcohols. These strains may play important roles in the production of fatty acid-based biofuels in cyanobacteria in future.

Compared to the heterotrophic bacteria such as *E. coli* and yeast, the current fatty alcohol productivity by cyanobacteria is still too low. Several additional approaches can be applied to improve *Synechocystis* engineering efficiency. First, heterologous gene expression can be improved by optimizing synonymous codon usage to better match the cyanobacterial host [42]. Second, the fatty alcohol biosynthesis can be enhanced by introducing more metabolic flux. Blocking the competing pathway of the PHB synthesis and the glycogen synthesis can drive the metabolic flux towards fatty alcohol synthesis [39]. Atsumi *et al.* showed overexpression of ribulose 1,5-bisphosphate carboxylase/oxygenase (RuBisCO) improves cyanobacterial isobutyraldehyde and isobutanol alcohol biosynthesis [43]. It would also be beneficial to the synthesis of long chain fatty alcohols. Third, the cultivation condition may also be improved by using higher light intensity (in the late growth phase) and higher CO_2_ concentration [15,43]. In a large scale and higher cell-density culture, light and CO_2_ can be limiting factors for cyanobacterial biosynthesis. Thereby, mixotrophic conditions may be an alternative solution. For example, *Synechocystis* can uptake acetate for acetyl-CoA synthesis [44] and improve product yield, and thus cheap carbon substrate can be employed for photo-biorefinery.

## Conclusions

In this study, the biosynthesis of fatty alcohols was achieved by expressing a fatty acyl-CoA reductase gene *maqu_2220* from *M. aquaeolei* VT8. The double knockout of the native *sll0208* and *sll0209* involved in the alkane biosynthesis pathway in *Synechocystis* improved the production of fatty alcohols. The results indicate that the deletion of the competing pathways for fatty alcohol biosynthesis could significantly improve the product yield.

The diverse fatty acyl-CoA (fatty acyl-ACP) reductases (Additional file 3: Table S1, Additional file 1: Figure S1) display a huge potential to molecularly modulate fatty alcohol product with the desired chain length and saturation degree, and provides feasibility to achieve the desired production of fatty alcohol-based biofuels and biochemicals. Further modifications of the metabolic network toward fatty acid biosynthesis are still needed for the significant improvement and application of photosynthetic fatty alcohol production in cyanobacteria.

## Methods

### Plasmid and strain construction

The prokaryotic fatty acyl-CoA reductase gene *maqu_2220* from *M. aquaeolei* VT8 (DSM 11845, purchased from Deutsche Sammlung von Mikroorganismen und Zellkulturen) was amplified by PCR using the primer pair faldr-1 and faldr-2 (Additional file 8: Table S2). It was sub-cloned into the cloning vector pMD18-T (Takara Co., Ltd, Kyoto, Japan) resulting in pXT71. The *maqu_2220* fragment in pXT71 was digested with *Nde*I/*Xho*I (Takara Co., Ltd, Kyoto, Japan) and ligated to the same site of pXT37b [15] generating the expression vector pFQ52. Then pFQ52 was transformed into the wild-type strain of *Synechocystis*. Transformation of *Synechocystis* was performed as described previously [15].

The plasmid pD0208 was constructed to disrupt the *sll0208* gene via homologous recombination in *Synechocystis.* A 2.4 kb of DNA fragment, which includes the open reading frame (ORF) and the upstream and downstream flanking regions of *sll0208*, was PCR-amplified from the genomic DNA of *Synechocystis* using primers D0208-F and D0208-R. This fragment was cloned into pGEM-T easy vector (Promega, USA) generating pGEMT-D0208. The plasmid pGEMT-D0208 was digested with *Hind* III (Takara Co., Ltd, Kyoto, Japan), blunted by T4 DNA polymerase (Thermo Scientific, USA) and ligated with the kanamycin resistance gene cassette C.K2 [45], which was amplified from the pRL446 plasmid [45] using primers C.K2-NF and C.K2-NR (Additional file 8: Table S2), generating the plasmid pD0208. The plasmid pLY76 was constructed to disrupt both the *sll0208* and *sll0209* gene in *Synechocystis*. Two 1.1 kb DNA fragments that included the upstream flanking region of *sll0208* and the downstream flanking region of *sll0209* were amplified using primer pairs D0809-1/D0809-2 and D0809-3/D0809-4 (Additional file 8: Table S2), respectively. The two DNA fragments containing a 33-bp overlapping region were used as templates to amplify a 2.3 kb DNA fragment. The fused fragment was sub-cloned into pMD18-T generating pLY75. The C.K2 cassette from the pRL446 plasmid [45] was sub-cloned into the blunted *Bam* HI site of pLY75 to generate the plasmid pLY76. The plasmid pD0208 and pLY76 were transformed into *Synechocystis* for homologous integration. The single knockout of the *sll0208* gene and the double knockout of *sll0208* and *sll0209* were verified by PCR.

### *Synechocystis* culture conditions

All *Synechocystis* strains were cultured at 30°C in 500 mL flasks containing 450 mL BG11 medium bubbled with air under constant fluorescent light at an intensity of 30 to 50 μE m^−2^ s^−1^. Cells were grown for 16 days in total and were sampled at 140, 274, and 384 hours for the measuring of fatty alcohols. Spectinomycin (20 μg mL^−1^) and/or kanamycin (20 μg mL^−1^) (Solarbio, Beijing, China) was added to the medium when needed. The correlation between the cell dry weight (DW) and optical density (OD) of the *Synechocystis* cells was confirmed by measuring both the OD_730_ and the cell Dry Weight (See Additional file 9: Figure S6 and Additional file 10: Table S3).

### Lipids extraction and GC–MS analysis

*Synechocystis* cells from 150 mL of culture were harvested by centrifugation, re-suspended in 10 mL of TE buffer (10 mM Tris-HCl, 1 mM EDTA, pH 8.0) and then lysed by sonication. The lysate was extracted for 30 minutes at room temperature with 10 mL chloroform-methanol (v/v, 2:1). Prior to extraction, 30 μg 1-pentadecanol, 50 μg eicosane, and 100 μg heptadecanoic acid (Sigma Aldrich, USA) were added into the cell lysate as internal standards (Figure 4). The organic phase was separated by centrifugation and evaporated to dryness under nitrogen at 55°C. The dryness was dissolved in 1 mL of n-hexane and 1-μL aliquot was analyzed by GC-MS using an Agilent 7890A system equipped with a HP-INNOWax (30 m × 250 μm × 0.25 μm, Agilent, USA). Helium (constant flow 1 mL min^−1^) was used as carrier gas. The temperature of the injector was 250°C. The following temperature program was applied: 100°C for 1 minute, increase of 5°C min^−1^ to 150°C and then 10°C min^−1^ to 250°C, hold at 250°C for 15 minutes. The internal standards were used to determine the product yield, which was the mean of three independent experiments [15].

## Abbreviations

AAR: acyl-ACP reductase; ACP: Acyl carrier protein; ADO: Aldehyde-deformylating oxygenase; DW: dry weight; FAR: Fatty acyl-CoA reductase; FFA: Free fatty acid; GC-MS: Gas chromatography-mass spectrometry. OD, optical density; PHB: poly-3-hydroxybutyrate.

## Competing interests

The authors declare that they have no competing interests.

## Authors’ contributions

LY and FQ carried out the construction and cultivation of *Synechocystis* sp. PCC 6803 mutant strains, extraction and analysis of free fatty acids, fatty alka(e)nes and fatty alcohols. LY, FQ and XL designed the experiments. LY and XL drafted the manuscript. XT and FQ participated in the design of the study and revised the manuscript. XL conceived of the study. All authors read and approved the final manuscript.

## Supplementary Material

Additional file 1: Table S1 The reported fatty acyl-CoA (or acyl-ACP) reductases in different organisms. Click here for file

Additional file 2: Figure S1 Phylogenetic relationships of the reported fatty acyl-CoA (or acyl-ACP) reductases. Click here for file

Additional file 3 **Nucleotide sequences of *maqu_2220 *from *Marinobacter aquaeolei VT8 *and P**_**petE **_**from *Synechocystis *sp. PCC 6803.** Click here for file

Additional file 4: Figure S2 PCR analysis of the mutant strains. Click here for file

Additional file 5: Figure S3 Contents and composition of fatty acid derivatives in the mutant and the wild-type *Synechocystis*. Click here for file

Additional file 6: Figure S4 Detection of fatty aldehyde in the mutant strains using GC-MS. Click here for file

Additional file 7: Figure S5 Experimental confirmation of the stability of fatty aldehyde to air and lipid extraction procedure. Click here for file

Additional file 8: Table S2 Primers used in PCR analysis. Click here for file

Additional file 9: Figure S6 The correlation between the dry cell weight (DCW) and optical density of the *Synechocystis* cells. Click here for file

Additional file 10: Table S3 Dry cell weight conversion factors of the five strains in this study. Click here for file

## References

[B1] Dellomonaco C Fava F Gonzalez R The path to next generation biofuels: successes and challenges in the era of synthetic biology Microb Cell Fact 2010 9 3 20089184 10.1186/1475-2859-9-3 PMC2817670

[B2] Lee SK Chou H Ham TS Lee TS Keasling JD Metabolic engineering of microorganisms for biofuels production: from bugs to synthetic biology to fuels Curr Opin Biotech 2008 19 556 563 18996194 10.1016/j.copbio.2008.10.014

[B3] Rude MA Schirmer A New microbial fuels: a biotech perspective Curr Opin Microbiol 2009 12 274 281 19447673 10.1016/j.mib.2009.04.004

[B4] Fortman JL Chhabra S Mukhopadhyay A Chou H Lee TS Steen E Keasling JD Biofuel alternatives to ethanol: pumping the microbial well Trends Biotechnol 2008 26 375 381 18471913 10.1016/j.tibtech.2008.03.008

[B5] Zhang FZ Rodriguez S Keasling JD Metabolic engineering of microbial pathways for advanced biofuels production Curr Opin Biotech 2011 22 775 783 21620688 10.1016/j.copbio.2011.04.024

[B6] Lu X A perspective: photosynthetic production of fatty acid-based biofuels in genetically engineered cyanobacteria Biotechnol Adv 2010 28 742 746 20561924 10.1016/j.biotechadv.2010.05.021

[B7] Parmar A Singh NK Pandey A Gnansounou E Madamwar D Cyanobacteria and microalgae: a positive prospect for biofuels Bioresour Technol 2011 102 10163 10172 21924898 10.1016/j.biortech.2011.08.030

[B8] Shih PM Wu DY Latifi A Axen SD Fewer DP Talla E Calteau A Cai F de Marsac NT Rippka R Herdman M Sivonen K Coursin T Laurent T Goodwin L Nolan M Davenport KW Han CS Rubin EM Eisen JA Woyke T Gugger M Kerfeld CA Improving the coverage of the cyanobacterial phylum using diversity-driven genome sequencing Proc Natl Acad Sci U S A 2013 110 1053 1058 23277585 10.1073/pnas.1217107110 PMC3549136

[B9] Angermayr SA Hellingwerf KJ Lindblad P de Mattos MJT Energy biotechnology with cyanobacteria Curr Opin Biotech 2009 20 257 263 19540103 10.1016/j.copbio.2009.05.011

[B10] Ducat DC Way JC Silver PA Engineering cyanobacteria to generate high-value products Trends Biotechnol 2011 29 95 103 21211860 10.1016/j.tibtech.2010.12.003

[B11] Deng MD Coleman JR Ethanol synthesis by genetic engineering in cyanobacteria Appl Environ Microbiol 1999 65 523 528 9925577 10.1128/aem.65.2.523-528.1999 PMC91056

[B12] Lindberg P Park S Melis A Engineering a platform for photosynthetic isoprene production in cyanobacteria, using *Synechocystis* as the model organism Metab Eng 2010 12 70 79 19833224 10.1016/j.ymben.2009.10.001

[B13] Atsumi S Higashide W Liao JC Direct photosynthetic recycling of carbon dioxide to isobutyraldehyde Nat Biotechnol 2009 27 1177 U1142 19915552 10.1038/nbt.1586

[B14] Shestakov SV Mikheeva LE Genetic control of hydrogen metabolism in cyanobacteria Russ J Genet 2006 42 1272 1284 17163069

[B15] Tan XM Yao L Gao QQ Wang WH Qi FX Lu XF Photosynthesis driven conversion of carbon dioxide to fatty alcohols and hydrocarbons in cyanobacteria Metab Eng 2011 13 169 176 21220042 10.1016/j.ymben.2011.01.001

[B16] Lan E Liao J Metabolic engineering of cyanobacteria for 1-butanol production from carbon dioxide Metab Eng 2011 13 353 363 21569861 10.1016/j.ymben.2011.04.004

[B17] Liu X Sheng J Curtiss R 3rd Fatty acid production in genetically modified cyanobacteria Proc Natl Acad Sci U S A 2011 108 6899 6904 21482809 10.1073/pnas.1103014108 PMC3084101

[B18] Niederholtmeyer H Wolfstadter BT Savage DF Silver PA Way JC Engineering cyanobacteria to synthesize and export hydrophilic products Appl Environ Microbiol 2010 76 6023 6023 10.1128/AEM.00202-10 PMC2876443 20363793

[B19] Dexter J Fu PC Metabolic engineering of cyanobacteria for ethanol production Energ Environ Sci 2009 2 857 864

[B20] Reinsvold RE Jinkerson RE Radakovits R Posewitz MC Basu C The production of the sesquiterpene beta-caryophyllene in a transgenic strain of the cyanobacterium *Synechocystis* J Plant Physiol 2011 168 848 852 21185107 10.1016/j.jplph.2010.11.006

[B21] Ducat DC Avelar-Rivas JA Way JC Silver PA Rerouting carbon flux to enhance photosynthetic productivity Appl Environ Microbiol 2012 78 2660 2668 22307292 10.1128/AEM.07901-11 PMC3318813

[B22] Kaczmarzyk D Fulda M Fatty acid activation in cyanobacteria mediated by acyl-acyl carrier protein synthetase enables fatty acid recycling Plant Physiol 2010 152 1598 1610 20061450 10.1104/pp.109.148007 PMC2832271

[B23] Gao Q Wang W Zhao H Lu X Effects of fatty acid activation on photosynthetic production of fatty acid-based biofuels in *Synechocystis sp.* PCC6803 Biotechnol Biofuels 2012 5 17 22433663 10.1186/1754-6834-5-17 PMC3366867

[B24] Schirmer A Rude MA Li X Popova E del Cardayre SB Microbial Biosynthesis of Alkanes Science 2010 329 559 562 20671186 10.1126/science.1187936

[B25] Danika T Bjorn V Annegret W Salim A Wolfgang RH Microevolution in Cyanobacteria: Re-sequencing a Motile Substrain of *Synechocystis sp* PCC 6803 DNA Res 2012 19 435 448 23069868 10.1093/dnares/dss024 PMC3514855

[B26] Trautmann D Beyer P Al-Babili S The ORF *slr0091* of *Synechocystis sp.* PCC6803 encodes a high-light induced aldehyde dehydrogenase converting apocarotenals and alkanals FEBS J 2013 280 3685 3696 23734995 10.1111/febs.12361

[B27] Metz JG Pollard MR Anderson L Hayes TR Lassner MW Purification of a *jojoba embryo* fatty acyl-coenzyme A reductase and expression of its cDNA in high erucic acid rapeseed Plant Physiol 2000 122 635 644 10712526 10.1104/pp.122.3.635 PMC58898

[B28] Rowland O Zheng HQ Hepworth SR Lam P Jetter R Kunst L CER4 encodes an alcohol-forming fatty acyl-coenzyme A reductase involved in cuticular wax production in *Arabidopsis* Plant Physiol 2006 142 866 877 16980563 10.1104/pp.106.086785 PMC1630741

[B29] Doan TTP Carlsson AS Hamberg M Bulow L Stymne S Olsson P Functional expression of five *Arabidopsis* fatty acyl-CoA reductase genes in *Escherichia coli* J Plant Physiol 2008 166 787 796 19062129 10.1016/j.jplph.2008.10.003

[B30] Cheng JB Russell DW Mammalian wax biosynthesis - I. Identification of two fatty acyl-coenzyme A reductases with different substrate specificities and tissue distributions J Biol Chem 2004 279 37789 37797 15220348 10.1074/jbc.M406225200 PMC2757098

[B31] Vioque J Kolattukudy PE Resolution and purification of an aldehyde-generating and an alcohol-generating fatty acyl-CoA reductase from pea leaves (*Pisum sativum L*) Arch Biochem Biophys 1997 340 64 72 9126278 10.1006/abbi.1997.9932

[B32] Teerawanichpan P Qiu X Fatty Acyl-CoA Reductase and Wax Synthase from Euglena gracilis in the Biosynthesis of Medium-Chain Wax Esters Lipids 2010 45 263 273 20195781 10.1007/s11745-010-3395-2

[B33] Willis RM Wahlen BD Seefeldt LC Barney BM Characterization of a fatty acyl-CoA reductase from *Marinobacter aquaeolei* VT8: a bacterial enzyme catalyzing the reduction of fatty acyl-CoA to fatty alcohol Biochemistry 2011 50 10550 10558 22035211 10.1021/bi2008646

[B34] Reiser S Somerville C Isolation of mutants of *Acinetobacter calcoaceticus* deficient in wax ester synthesis and complementation of one mutation with a gene encoding a fatty acyl coenzyme a reductase J Bacteriol 1997 179 2969 2975 9139916 10.1128/jb.179.9.2969-2975.1997 PMC179062

[B35] Wahlen BD Oswald WS Seefeldt LC Barney BM Purification, characterization, and potential bacterial wax production role of an NADPH-dependent fatty aldehyde reductase from *Marinobacter aquaeolei* VT8 Appl Environ Microbiol 2009 75 2758 2764 19270127 10.1128/AEM.02578-08 PMC2681700

[B36] Hofvander P Doan TTP Hamberg M A prokaryotic acyl-CoA reductase performing reduction of fatty acyl-CoA to fatty alcohol FEBS Lett 2011 585 3538 3543 22020216 10.1016/j.febslet.2011.10.016

[B37] Liu A Tan X Yao L Lu X Fatty alcohol production in engineered *E. coli* expressing *Marinobacter* fatty acyl-CoA reductases Appl Microbiol Biotechnol 2013 97 7061 7071 23793343 10.1007/s00253-013-5027-2

[B38] Runguphan W Keasling JD Metabolic engineering of *Saccharomyces cerevisiae* for production of fatty acid-derived biofuels and chemicals Metab Eng 2014 21 103 113 23899824 10.1016/j.ymben.2013.07.003

[B39] Qi F Yao L Tan X Lu X Construction, characterization and application of molecular tools for metabolic engineering of *Synechocystis sp* Biotechnol Lett 2013 35 1655 1661 23743956 10.1007/s10529-013-1252-0

[B40] Prentki P Binda A Epstein A Plasmid vectors for selecting IS1-promoted deletions in cloned DNA: sequence analysis of the omega interposon Gene 1991 103 17 23 1652541 10.1016/0378-1119(91)90385-o

[B41] Kaiser BK Carleton M Hickman JW Miller C Lawson D Budde M Warrener P Paredes A Mullapudi S Navarro P Cross F Roberts JM Fatty aldehydes in cyanobacteria are a metabolically flexible precursor for a diversity of biofuel products PLoS One 2013 8 e58307 23505484 10.1371/journal.pone.0058307 PMC3594298

[B42] Varman AM Yu Y You L Tang YJ Photoautotrophic production of D-lactic acid in an engineered cyanobacterium Microb Cell Fact 2013 12 117 24274114 10.1186/1475-2859-12-117 PMC4222570

[B43] Atsumi S Higashide W Liao JC Direct photosynthetic recycling of carbon dioxide to isobutyraldehyde Nat Biotech 2009 27 1177 1180 10.1038/nbt.1586 19915552

[B44] Wu G Bao T Shen Z Wu Q Sodium acetate stimulates PHB biosynthesis in *Synechocystis sp*. PCC 6803 Tsinghua Sci Technol 2002 7 435 438

[B45] Xu XD Yin CT Li WZ Du Y Kong RQ Identification of a gene, *ccr-1* (*sll1242*), required for chill-light tolerance and growth at 15 degrees C in *Synechocystis sp* PCC 6803 Microbiol-Sgm 2007 153 1261 1267 10.1099/mic.0.2006/005074-0 17379735

