# Supplementary material for: Improved production of fatty alcohols in cyanobacteria by metabolic engineering
Source: Biotechnol Biofuels. 2014 Jun 18;7:94. doi: 10.1186/1754-6834-7-94 (PMC4096523; doi:10.1186/1754-6834-7-94)
Supplement: Additional file 1: Table S1 — The reported fatty acyl-CoA (or acyl-ACP) reductases in different organisms. [file 1754-6834-7-94-S1.docx]

**Table S1 The reported fatty acyl-CoA (or acyl-ACP) reductases in different organisms.**

| **Gene name** | **Host organism** | **Cofactor** | **Primary substrate specificity^#^** | **GenBank accession no.** | **References** |
| --- | --- | --- | --- | --- | --- |
| *AaTFAR1^a^* | *Artemisia annua* | U | U | GU733320 | [[1](#_ENREF_1)] |
| *acr1^b^* | *Acinetobacter calcoaceticus* | NADPH | C14:0, C16:0, C18:0 | U77680 | [[2](#_ENREF_2)] |
| *AdFAR1 ^a^* | *Anser anser domesticus* | NADPH | C14:0, C16:0, C18:0 | JN638548 | [[3](#_ENREF_3)] |
| *AmFAR1 ^a^* | *Apis mellifera* | NADPH | C18:0, C20:0, C22:0 | HM483391 | [[4](#_ENREF_4)] |
| *AtCER4 ^a^* | *Arabidopsis thaliana* | U | C24:0, C26:0, C28:0, C30:0 | AY070065 | [[5](#_ENREF_5)] |
| *AtFAR1 ^a^* |  | U | C22:0 | EU280149 | [[6](#_ENREF_6)] |
| *AtFAR4 ^a^* |  | U | C20:0 | AK227396 | [[6](#_ENREF_6)] |
| *AtFAR5 ^a^* |  | U | C18:0 | AK228404 | [[6](#_ENREF_6)] |
| *AtFAR6 ^a^* |  | NADPH | C16:0 | EU280151 | [[7](#_ENREF_7)] |
| *At*[*FAR8*](http://www.ncbi.nlm.nih.gov/gene/823581) *^a^* |  | U | U | EU280153 | [[8](#_ENREF_8)] |
| *AtMS2 ^a^* |  | NADPH / NADH | C16:0 | EU280150 | [[9](#_ENREF_9)] |
| *BbFAR ^b^* | *Botryococcus braunii* | NADH | U | None | [[10](#_ENREF_10)] |
| *BmFAR ^a^* | *Bombyx mori* | NADPH | C15:0, C16:0, C16:1, C16:2 | AB104896 | [[11](#_ENREF_11)] |
| *CfFAR1 ^a^* | *Calanus finmarchicus* | NADPH | C18:0, C20:0, C22:0, C24:0, C26:0 | JN243755 | [[12](#_ENREF_12)] |
| *CfFAR2 ^a^* |  | NADPH | C24:0, C26:0 | JN243756 | [[12](#_ENREF_12)] |
| *CfFAR3 ^a^* |  | NADPH | C16:0, C18:0, C18:1, C20:1 | JN243757 | [[12](#_ENREF_12)] |
| *EgFAR ^a^* | *Euglena gracilis* | NADH | C14:0, C16:0 | GU733919 | [[13](#_ENREF_13)] |
| *GgFAR1 ^a^* | *Gallus gallus* | NADPH | C16:0, C18:0 | NM_001031179 | [[14](#_ENREF_14)] |
| *GgFAR2 ^a^* |  | NADPH | C18:0 | XM_417235 | [[3](#_ENREF_3)] |
| *HsFAR1 ^a^* | *Homo sapiens* | U | U | AY600449 | [[15](#_ENREF_15)] |
| *HsFAR2 ^a^* |  | U | U | BC022267 | [[15](#_ENREF_15)] |
| *MaFAR ^a^* | *Marinobacter aquaeolei* VT8 | NADPH | C14:0, C16:0, C16:1, C18:0, C18:1, C20:4 | YP_959769 | [[16](#_ENREF_16)] |
| *Maqu_2220 ^a^* |  | NADPH | C14:0, C16:0, C18:0, C18:1, C20:0 | ABM19299 | [[17](#_ENREF_17)] |
| *MmFAR1 ^a^* | *Mus musculus* | NADPH | C16:0, C18:0, C18:1, C18:2 | BC007178 | [[15](#_ENREF_15)] |
| *MmFAR2 ^a^* |  | NADPH | C16:0, C18:0 | BC055759 | [[15](#_ENREF_15)] |
| *OnpgFAR-E ^a^* | *Ostrinia nubilalis* | U | U | FJ807735 | [[18](#_ENREF_18)] |
| *OnpgFAR-Z ^a^* |  | U | U | FJ807736 | [[18](#_ENREF_18)] |
| *orf1594 ^b^* | [*Synechococcus* elongatus PCC 7942](http://microbes.ucsc.edu/cgi-bin/hgGateway?db=syneElon_PCC_7942) | NADPH | U | YP_400611 | [[19](#_ENREF_19)] |
| *OsFAR ^a^* | *Oryza sativa* | NADPH | C16:0, C16:1 | AK121254 | [[20](#_ENREF_20)] |
| *OsFARXIII ^a^* | *Ostrinia scapulalis* | U | C14:1 | EU817405 | [[21](#_ENREF_21)] |
| *Pp-luxC ^b^* | *Photobacterium phosphoreum* | NADPH | U | L21989 | [[22](#_ENREF_22)] |
| *PsFAR1 ^a^* | *Pisum sativum* | NADPH / NADH | U | None | [[23](#_ENREF_23)] |
| *PsFAR2 ^b^* |  | NADPH / NADH | U | None | [[23](#_ENREF_23)] |
| *ScFAR ^a^* | *Simmondsia chinensis* | NADPH | C20:1, C22:1, C24:1 | AF149917 | [[24](#_ENREF_24)] |
| *sll0209 ^b^* | *Synechocystis* sp. PCC6803 | U | U | NP_442146 | [[19](#_ENREF_19)] |
| *TaFAR1 ^a^* | *Tyto alba* | NADPH | C14:0, C16:0 | JN638549 | [[14](#_ENREF_14)] |
| *TaFAR2 ^a^* |  | NADPH | C18:0 | JN638550 | [[3](#_ENREF_3)] |
| *TaTAA1a ^a^* | *Triticum aestivum* | U | C18:1, C20:1, C22:1, C24:0, C26:0 | AJ459249 | [[25](#_ENREF_25)] |
| *YepgFA ^a^ R* | *Yponomeuta evonymellus* | U | C14:0, C14:1, C16:0, C16:1 | GQ907232 | [[26](#_ENREF_26)] |
| *YppgFAR ^a^* | *Yponomeuta padellus* | U | C14:0, C14:1, C16:0, C16:1 | GQ907235 | [[26](#_ENREF_26)] |
| *YrpgFAR ^a^* | *Yponomeuta rorrellus* | U | C14:0, C14:1, C16:0, C16:1 | GQ907234 | [[26](#_ENREF_26)] |

^*^Type A, four-electron reduction fatty acyl-CoA reductase generating fatty alcohol. Type B, two-electron reduction fatty acyl-CoA reductase generating fatty aldehyde.

^#^Substrate specificities of FARs were obtained from the in vitro studies of FAR enzymes or deduced from expression of FARs in native or heterologous systems.

# References

1. Maes L, Van Nieuwerburgh FCW, Zhang YS, Reed DW, Pollier J, Casteele S, Inze D, Covello PS, Deforce DLD, Goossens A: **Dissection of the phytohormonal regulation of trichome formation and biosynthesis of the antimalarial compound artemisinin in Artemisia annua plants.** *New Phytol* 2011, **189:**176-189.

2. Reiser S, Somerville C: **Isolation of mutants of Acinetobacter calcoaceticus deficient in wax ester synthesis and complementation of one mutation with a gene encoding a fatty acyl coenzyme a reductase.** *J Bacteriol* 1997, **179:**2969-2975.

3. Hellenbrand J, Biester E-M, Gruber J, Hamberg M, Frentzen M: **Fatty acyl-CoA reductases of birds.** *BMC Biochemistry* 2011, **12**.

4. Teerawanichpan P, Robertson AJ, Qiu XA: **A fatty acyl-CoA reductase highly expressed in the head of honey bee (Apis mellifera) involves biosynthesis of a wide range of aliphatic fatty alcohols.** *Insect Biochem Mol Biol* 2010, **40:**641-649.

5. Rowland O, Zheng HQ, Hepworth SR, Lam P, Jetter R, Kunst L: **CER4 encodes an alcohol-forming fatty acyl-coenzyme A reductase involved in cuticular wax production in Arabidopsis.** *Plant Physiol* 2006, **142:**866-877.

6. Domergue F, Vishwanath SJ, Joubes J, Ono J, Lee JA, Bourdon M, Alhattab R, Lowe C, Pascal S, Lessire R, Rowland O: **Three Arabidopsis Fatty Acyl-Coenzyme A Reductases, FAR1, FAR4, and FAR5, Generate Primary Fatty Alcohols Associated with Suberin Deposition.** *Plant Physiol* 2010, **153:**1539-1554.

7. Doan TTP, Domergue F, Fournier AE, Vishwanath SJ, Rowland O, Moreau P, Wood CC, Carlsson AS, Hamberg M, Hofvander P: **Biochemical characterization of a chloroplast localized fatty acid reductase from Arabidopsis thaliana.** *Biochimica Et Biophysica Acta-Molecular and Cell Biology of Lipids* 2012, **1821:**1244-1255.

8. Doan TTP, Carlsson AS, Hamberg M, Bulow L, Stymne S, Olsson P: **Functional expression of five Arabidopsis fatty acyl-CoA reductase genes in Escherichia coli.** *J Plant Physiol* 2008, **166:**787-796.

9. Chen WW, Yu XH, Zhang KS, Shi JX, De Oliveira S, Schreiber L, Shanklin J, Zhang DB: **Male Sterile2 Encodes a Plastid-Localized Fatty Acyl Carrier Protein Reductase Required for Pollen Exine Development in Arabidopsis.** *Plant Physiol* 2011, **157:**842-853.

10. Wang X, Kolattukudy PE: **SOLUBILIZATION AND PURIFICATION OF ALDEHYDE-GENERATING FATTY ACYL-COA REDUCTASE FROM GREEN-ALGA BOTRYOCOCCUS-BRAUNII.** *FEBS Lett* 1995, **370:**15-18.

11. Moto Ki, Yoshiga T, Yamamoto M, Takahashi S, Okano K, Ando T, Nakata T, Matsumoto S: **Pheromone gland-specific fatty-acyl reductase of the silkmoth, Bombyx mori.** *Proceedings of the National Academy of Sciences of the United States of America* 2003, **100:**9156-9161.

12. Teerawanichpan P, Qiu X: **Molecular and Functional Analysis of Three Fatty Acyl-CoA Reductases with Distinct Substrate Specificities in Copepod Calanus finmarchicus.** *Mar Biotechnol* 2012, **14:**227-236.

13. Teerawanichpan P, Qiu X: **Fatty Acyl-CoA Reductase and Wax Synthase from Euglena gracilis in the Biosynthesis of Medium-Chain Wax Esters.** *Lipids* 2010, **45:**263-273.

14. Hellenbrand J, Biester EM, Gruber J, Hamberg M, Frentzen M: **Fatty acyl-CoA reductases of birds.** *BMC Biochemistry* 2011, **12**.

15. Cheng JB, Russell DW: **Mammalian wax biosynthesis - I. Identification of two fatty acyl-coenzyme A reductases with different substrate specificities and tissue distributions.** *J Biol Chem* 2004, **279:**37789-37797.

16. Willis RM, Wahlen BD, Seefeldt LC, Barney BM: **Characterization of a Fatty Acyl-CoA Reductase from Marinobacter aquaeolei VT8: A Bacterial Enzyme Catalyzing the Reduction of Fatty Acyl-CoA to Fatty Alcohol.** *Biochemistry-Us* 2011, **50:**10550-10558.

17. Hofvander P, Doan TTP, Hamberg M: **A prokaryotic acyl-CoA reductase performing reduction of fatty acyl-CoA to fatty alcohol.** *FEBS Lett* 2011, **585:**3538-3543.

18. Lassance J-M, Groot AT, Lienard MA, Antony B, Borgwardt C, Andersson F, Hedenstrom E, Heckel DG, Lofstedt C: **Allelic variation in a fatty-acyl reductase gene causes divergence in moth sex pheromones.** *Nature* 2010, **466:**486-U487.

19. Schirmer A, Rude MA, Li X, Popova E, del Cardayre SB: **Microbial biosynthesis of alkanes.** *Science* 2010, **329:**559-562.

20. Shi J, Tan HX, Yu XH, Liu YY, Liang WQ, Ranathunge K, Franke RB, Schreiber L, Wang YJ, Kai GY, et al: **Defective Pollen Wall Is Required for Anther and Microspore Development in Rice and Encodes a Fatty Acyl Carrier Protein Reductase.** *Plant Cell* 2011, **23:**2225-2246.

21. Antony B, Fujii T, Moto K, Matsumoto S, Fukuzawa M, Nakano R, Tatsuki S, Ishikawa Y: **Pheromone-gland-specific fatty-acyl reductase in the adzuki bean borer, Ostrinia scapulalis (Lepidoptera: Crambidae).** *Insect Biochem Mol Biol* 2009, **39:**90-95.

22. Lee CY, Meighen EA: **Expression and DNA sequence of the gene coding for the lux-specific fatty acyl-CoA reductase from Photobacterium phosphoreum (vol 38, pg 80, 2000).** *J Microbiol* 2000, **38:**281-281.

23. Vioque J, Kolattukudy PE: **Resolution and purification of an aldehyde-generating and an alcohol-generating fatty acyl-CoA reductase from pea leaves (Pisum sativum L).** *Arch Biochem Biophys* 1997, **340:**64-72.

24. Metz JG, Pollard MR, Anderson L, Hayes TR, Lassner MW: **Purification of a jojoba embryo fatty acyl-coenzyme A reductase and expression of its cDNA in high erucic acid rapeseed.** *Plant Physiol* 2000, **122:**635-644.

25. Wang AM, Xia Q, Xie WS, Dumonceaux T, Zou JT, Datla R, Selvaraj G: **Male gametophyte development in bread wheat (Triticum aestivum L.): molecular, cellular, and biochemical analyses of a sporophytic contribution to pollen wall ontogeny.** *Plant J* 2002, **30:**613-623.

26. Lienard MA, Hagstrom AK, Lassance JM, Lofstedt C: **Evolution of multicomponent pheromone signals in small ermine moths involves a single fatty-acyl reductase gene.** *Proceedings of the National Academy of Sciences of the United States of America* 2010, **107:**10955-10960.
