# Supplementary material for: Improved production of fatty alcohols in cyanobacteria by metabolic engineering
Source: Biotechnol Biofuels. 2014 Jun 18;7:94. doi: 10.1186/1754-6834-7-94 (PMC4096523; doi:10.1186/1754-6834-7-94)
Supplement: Additional file 2: Figure S1 — Phylogenetic relationships of the reported fatty acyl-CoA (or acyl-ACP) reductases. [file 1754-6834-7-94-S2.docx]

**Figure S1 Phylogenetic relationships of the reported fatty acyl-CoA (or acyl-ACP) reductases.**

Trees were constructed by the neighbor joining method. The numbers at each branch point are the bootstrap values for the percentages of 1000 replicate trees; only values >50% are shown. The *Marinobacter aquaeolei* VT8 Maqu_2220 and the *Synechocystis* Sll0209 are in different colors. Among all the reported fatty acyl-CoA (or acyl-ACP) reductases (FARs), Maqu_2220 is the prokaryotic FAR that revealed close relationship to the cyanobacterial FARs. ^a^. The type A FARs that catalyse 4-electron reductions; ^b^. The type B FARs that catalyse 2-electron reductions.
