# Supplementary material for: Improved production of fatty alcohols in cyanobacteria by metabolic engineering
Source: Biotechnol Biofuels. 2014 Jun 18;7:94. doi: 10.1186/1754-6834-7-94 (PMC4096523; doi:10.1186/1754-6834-7-94)
Supplement: Additional file 3 — Nucleotide sequences of maqu_2220 from Marinobacter aquaeolei VT8 and PpetE from Synechocystis sp. PCC 6803. [file 1754-6834-7-94-S3.docx]

## Nucleotide sequences (5’-3’):

**>*****maqu_2220* from *Marinobacter aquaeolei* VT8**

ATGGCAATACAGCAGGTACATCACGCTGACACTTCATCATCAAAGGTGCTCGGACAGCTCCGTGGCAAGCGGGTTCTGATCACCGGTACCACTGGCTTTCTGGGCAAGGTGGTCCTCGAAAGGCTGATTCGGGCGGTGCCTGATATCGGCGCAATTTACCTGCTGATCCGGGGCAATAAACGGCATCCGGATGCTCGTTCCCGTTTCCTGGAAGAAATTGCCACCTCCTCGGTGTTTGACCGTCTTCGCGAGGCCGATTCAGAGGGATTTGACGCCTTTCTGGAAGAGCGCATTCACTGCGTGACCGGTGAGGTGACCGAAGCGGGTTTCGGGATAGGGCAGGAAGACTATCGCAAACTCGCCACCGAACTGGATGCGGTGATCAACTCCGCTGCAAGCGTGAATTTCCGTGAAGAGCTCGACAAGGCGCTGGCCATCAACACCCTGTGCCTTCGGAATATTGCCGGCATGGTGGATTTGAATCCGAAGCTTGCGGTCCTGCAGGTCTCCACCTGCTATGTCAATGGCATGAACTCGGGGCAGGTAACCGAATCGGTGATCAAGCCGGCAGGCGAGGCCGTGCCGCGTTCCCCGGACGGCTTCTATGAGATAGAAGAGCTTGTTCGCCTGCTTCAGGATAAAATTGAAGACGTTCAGGCCCGTTATTCCGGCAAAGTGCTGGAGAGGAAGCTGGTGGACCTGGGGATTCGGGAAGCCAACCGCTATGGCTGGAGCGATACCTACACCTTTACCAAGTGGCTGGGCGAACAGTTGCTGATGAAGGCGTTAAACGGGCGCACGCTGACCATTCTGCGTCCTTCGATTATCGAAAGTGCCCTGGAGGAACCAGCGCCCGGCTGGATTGAGGGGGTGAAGGTGGCAGATGCCATCATCCTGGCTTACGCACGGGAAAAAGTCACCCTCTTCCCGGGCAAACGCTCCGGTATCATCGATGTGATTCCAGTGGACCTGGTGGCCAACTCCATCATCCTTTCCCTGGCGGAAGCTCTTGGAGAACCCGGTCGACGTCGCATCTATCAATGTTGCAGCGGGGGCGGCAATCCAATCTCCCTGGGTGAGTTCATCGATCATCTCATGGCGGAATCAAAAGCCAATTACGCTGCCTACGATCACCTGTTCTACCGGCAGCCCAGCAAGCCGTTTCTGGCGGTTAACCGGGCGCTGTTTGATTTGGTGATCAGTGGTGTTCGCTTACCGCTCTCCCTGACGGACCGTGTGCTCAAATTACTGGGAAATTCCCGGGACCTGAAAATGCTCAGGAATCTGGATACCACCCAGTCGCTGGCAACCATTTTTGGTTTCTACACCGCGCCGGATTATATCTTCCGGAACGATGAGCTGATGGCGCTGGCGAACCGGATGGGTGAGGTCGATAAAGGGCTGTTCCCGGTGGATGCCCGCCTGATTGACTGGGAGCTCTACCTGCGCAAGATTCACCTGGCCGGGCTCAATCGCTATGCCCTGAAAGAACGAAAGGTGTACAGTCTGAAAACCGCGCGCCAGCGCAAAAAAGCTGCCTGA

**>PpetE**

AAGGATTCATAGCGGTTGCCCAATCTAACTCAGGGAGCGACTTCAGCCCACAAAAAACACCACTGGGCCTACTGGGCTATTCCCATTATCATCTACATTGAAGGGATAGCAAGCTAATTTTTATGACGGCGATCGCCAAAAACAAAGAAAATTCAGCAATTACCGTGGGTAGCAAAAAATCCCCATCTAAAGTTCAGTAAATATAGCTAGAACAACCAAGCATTTTCGGCAAAGTACTATTCAGATAGAACGAGAAATGAGCTTGTTCTATCCGCCCGGGGCTGAGGCTGTATAATCTACGACGGGCTGTCAAACATTGTGATACCATGGGCAGAAGAAAGGAAAAACGTCCCTGATCGCCTTTTTGGGCACGGAGTAGGGCGTTACCCCGGCCCGTTCAACCACAAGTCCCTATAGATACAATCGCCAAGAAGTATGTC
