# Supplementary material for: Improved production of fatty alcohols in cyanobacteria by metabolic engineering
Source: Biotechnol Biofuels. 2014 Jun 18;7:94. doi: 10.1186/1754-6834-7-94 (PMC4096523; doi:10.1186/1754-6834-7-94)
Supplement: Additional file 4: Figure S2 — PCR analysis of the mutant strains. [file 1754-6834-7-94-S4.docx]

**Figure S2 PCR analysis of the mutant strains.**

A, P_petE_-t and faldr-2 were used as primer pair to test whether the DNA constructs were introduced or not. B, faldr-1 and 0168-2 were used as primer pair to test whether the DNA constructs were introduced into *slr0168* locus. C, 0168-1 and 0168-2 were used as primer pair to test the complete segregation of the mutant strain. D, D0208-F and D0208-R were used as primer pair to test the complete disruption of *sll0208*. E, Dsll0209-1 and D0809-R were used as primer pair to test the complete disruption of *sll0208* and *sll0209*. Lane 1-3 (in A, B and C): PCR using genomic DNA from *Synechocystis* wild type (negative control), Syn-FQ52 and Syn-FQ52D08 as templates respectively. Lane 1-3(in D): PCR using genomic DNA from *Synechocystis* wild type (negative control), Syn-D08 and Syn-FQ52D08 as templates respectively. Lane 1-2 (in E): PCR using genomic DNA from *Synechocystis* wild type (negative control) and Syn-FQ52D0809 as templates respectively.
