# Supplementary material for: Improved production of fatty alcohols in cyanobacteria by metabolic engineering
Source: Biotechnol Biofuels. 2014 Jun 18;7:94. doi: 10.1186/1754-6834-7-94 (PMC4096523; doi:10.1186/1754-6834-7-94)
Supplement: Additional file 5: Figure S3 — Contents and composition of fatty acid derivatives in the mutant and the wild-type Synechocystis. [file 1754-6834-7-94-S5.docx]

**Figure S3 Contents and composition of fatty acid derivatives in the mutant and the wild type *Synechocystis*.**

A, Contents and composition of hydrocarbons in the mutant and the wild type *Synechocystis* grown for 140, 274 and 384 hours respectively. B, Contents and composition of free fatty acids in the mutant and the wild type *Synechocystis* grown for 140, 274 and 384 hours respectively. C, Contents and compositions of total fatty acid derivatives in different *Synechocystis* strains. D, The composition and total amount of fatty acyl chains (fatty alcohol, hydrocarbon and free fatty acid) in these *Synechocystis* strains.
