# Supplementary material for: Improved production of fatty alcohols in cyanobacteria by metabolic engineering
Source: Biotechnol Biofuels. 2014 Jun 18;7:94. doi: 10.1186/1754-6834-7-94 (PMC4096523; doi:10.1186/1754-6834-7-94)
Supplement: Additional file 6: Figure S4 — Detection of fatty aldehyde in the mutant strains using GC-MS. [file 1754-6834-7-94-S6.docx]

**Figure S4 Detection of fatty aldehyde in the mutant strains using GC-MS.**

The extracts from the mutant strains were analyzed using GC-MS. The hexadecanal and octadecanal were chosen as the external fatty aldehyde standards and went through the same GC-MS program. The result shows no hexadecanal or octadecanal was detected in the wild type and the mutant *Synechocystis* strains.
