# Supplementary material for: Improved production of fatty alcohols in cyanobacteria by metabolic engineering
Source: Biotechnol Biofuels. 2014 Jun 18;7:94. doi: 10.1186/1754-6834-7-94 (PMC4096523; doi:10.1186/1754-6834-7-94)
Supplement: Additional file 7: Figure S5 — Experimental confirmation of the stability of fatty aldehyde to air and lipid extraction procedure. [file 1754-6834-7-94-S7.docx]

**Figure S5 Experimental confirmation of the stability of fatty aldehyde to air and lipid extraction procedure.**

To test the fatty aldehyde oxidation during the experimental conditions, the stability of fatty aldehyde to open air and the fat extract procedures was analyzed using GC. A pure fatty aldehyde solution of hexadecanal and octadecanal were exposed to open air for 1, 2 or 10 hours before analysis. The result shows that hexadecanal and octadecanal were quite stable and not oxidized into fatty acids in 10 hours. To test the influence from lipid extract process, two concentration of hexadecanal and octadecanal solution (50 g L^-1^ and 200 g L^-1^) were prepared and treated with the chloroform and methanol extraction method; a control (Water extraction) for lipid extraction that using water instead of the cell culture was also prepared. In the two samples, the abundance of fatty aldehyde decreased after the extraction process, however, the lipid extraction process led to none fatty acids accumulation compared to the control. FFA: free fatty acids; C16:0 FFA: hexadecanoic acid; C18:0 FFA: Octadecanoic acid; 1-hour air exposition: fatty aldehyde solution exposed to open air for 1 hour.
