# Supplementary material for: Improved production of fatty alcohols in cyanobacteria by metabolic engineering
Source: Biotechnol Biofuels. 2014 Jun 18;7:94. doi: 10.1186/1754-6834-7-94 (PMC4096523; doi:10.1186/1754-6834-7-94)
Supplement: Additional file 8: Table S2 — Primers used in PCR analysis. [file 1754-6834-7-94-S8.docx]

**Table S2 Primers used in PCR analysis.**

| Primer | Sequence (5' to 3') |
| --- | --- |
| faldr-1 | TCATATGGCAATACAGCAGGTACATCA |
| faldr-2 | ACTCGAGTCAGGCAGCTTTTTTGCGC |
| D0208-F | CTTGATTCTACTGCGGCGAG |
| D0208-R | CCAACGACATACGGCGCTAC |
| CK2-NF | CCATGGTGTGCTGCAAGGCGATTAAG |
| CK2-NR | CCATGGTCCGGCTCGTATGTTGTGTG |
| P_petE_-t | CCGTTCAACCACAAGTCCCTAT |
| 0168-1 | ACCTCTCCACGCTGAATTAG |
| 0168-2 | TCCAGGCCACATTGTTGTC |
| D0809-1 | TAACCTAATTCAAGACCACCTAGAGC |
| D0809-2 | CACGCAGTCGACTTCACACTCGGATCCTATAACCAATGACAATGGCGTTGATGC |
| D0809-3 | GTTATAGGATCCGAGTGTGAAGTCGACTGCGTGTAGACACCAGGGTGAAAGCG |
| D0809-4 | GCCTACTTAGCGGTGATTGG |
| Dsll0209-1 | GAATGGTGCCAACTCAAATCGC |
| D0809-R | GTGGAGATGGATATTCCCTCCC |
