# Supplementary material for: Improved production of fatty alcohols in cyanobacteria by metabolic engineering
Source: Biotechnol Biofuels. 2014 Jun 18;7:94. doi: 10.1186/1754-6834-7-94 (PMC4096523; doi:10.1186/1754-6834-7-94)
Supplement: Additional file 9: Figure S6 — The correlation between the dry cell weight (DCW) and optical density of the Synechocystis cells. [file 1754-6834-7-94-S9.docx]

**Figure S6 The** **correlation between the dry cell weight (DCW) and optical density of the *Synechocystis* cells.**

To calculate the dry cell weights of the five *Synechocystis* strains shown in the above figure, 20 mL cell culture of each strain at five time points were prepared. All samples were collected in triplicate at each time point. Each time point is the average dry cell weight of the triplicates. Error bars represent the standard error from the mean. OD_730_: the optical density of *Synechocystis* culture at 730 nm.
