# Supplementary material for: Improved production of fatty alcohols in cyanobacteria by metabolic engineering
Source: Biotechnol Biofuels. 2014 Jun 18;7:94. doi: 10.1186/1754-6834-7-94 (PMC4096523; doi:10.1186/1754-6834-7-94)
Supplement: Additional file 10: Table S3 — Dry cell weight conversion factors of the five strains in this study. [file 1754-6834-7-94-S10.docx]

## Table S3 Dry cell weight conversion factors of the five strains in this study.

| Strains | 6803WT | Syn-D08 | Syn-FQ52 | Syn-FQ52D08 | Syn-FQ52D0809 |
| --- | --- | --- | --- | --- | --- |
| dry cell weight  (mg OD_730_^-1^L^-1^) | 187.76 | 194.49 | 189.76 | 167.68 | 173.31 |

6803WT: the wild-type strain of *Synechocystis*; Syn-D08: the strain with deletion of *sll0208*; Syn-FQ52: the strain with expression of *maqu_2220*; Syn-FQ52D08: the strain with deletion of *sll0208* and expression of *maqu_2220*; Syn-FQ52D0809: the strain with deletion of *sll0208* and *sll0209*, and expression of *maqu_2220*.
